# Origin of water in the Moon

**DOI:** 10.1093/nsr/nwae151

**Published:** 2024-04-29

**Authors:** Hejiu Hui, Ziyan Han, Kang Shuai

**Affiliations:** State Key Laboratory of Mineral Deposits Research & Lunar and Planetary Science Institute, School of Earth Sciences and Engineering, Nanjing University, China; CAS Center for Excellence in Comparative Planetology, China; CAS Key Laboratory of Earth and Planetary Physics, Institute of Geology and Geophysics, China; State Key Laboratory of Mineral Deposits Research & Lunar and Planetary Science Institute, School of Earth Sciences and Engineering, Nanjing University, China; State Key Laboratory of Mineral Deposits Research & Lunar and Planetary Science Institute, School of Earth Sciences and Engineering, Nanjing University, China

It has been well known since the Apollo era that the Moon is relatively depleted in volatiles as a result of the Moon-formation impact and the subsequent planetary differentiation. The lunar interior was believed to have been extremely dry [1]. The discovery of indigenous water (a collective term for H-bearing species OH, H_2_O, etc.) in various lunar materials [2–5] has driven a paradigm shift in our knowledge of water in the Moon, from a dry to a wet Moon. These discoveries have invoked investigations on the consequences of indigenous water in the lunar interior for the origin of the Moon in the context of giant-impact formation theory [6–8]. The origin of this indigenous water in the lunar interior has become key to understanding the formation of the Moon.

The large variation in hydrogen isotope composition in mare apatite (–200‰ to +1430‰) was originally interpreted as water in lunar rocks that may have originated from lunar mantle (–280‰ to +187‰), solar wind (approximately –1000‰) and comet (+33‰ to +1600‰) [9–12]. The implantation of solar wind protons on the Moon's surface could have hydrated the surface materials with extremely low δD (–990‰) [13]. On the other hand, mare basalts could have assimilated low-melting-point regolith particles on the Moon's surface during magma emplacement [14]. Hence, the incorporation of surface materials into the mare magma could have led to the presence of solar wind proton signature in late-stage apatite of mare basalts [15]. Furthermore, magma degassing during mare basalt eruption could have resulted in high δD (up to +1430‰) in mare apatite [10,12]. In other words, the high δD observed in mare apatite may have resulted from extensive magma degassing and thus may not necessarily represent a comet reservoir for water in lunar materials. Note that experimental determination of hydrogen isotope fractionation under lunar conditions is still lacking. By combining the available hydrogen isotope data in the context of the lunar magma ocean (LMO) hypothesis, a model on the early evolution of hydrogen isotopes in the Moon has been proposed, suggesting the existence of reservoirs in the lunar interior with different hydrogen compositions, both elemental and isotopic [12]. This model could account for the large δD variations recorded in mare apatites of up to +1430‰, as well as the δD values observed in volcanic glasses (+30‰ to +770‰) and highlands phases (–280‰ to +340‰) [10,12]. More importantly, the LMO may have had an initial hydrogen isotope composition of about –280‰ [12].

This initial LMO hydrogen isotope composition is similar to δD of Earth's deep primordial mantle (–200‰ ± 50‰) [12,16,17]. This similarity is consistent with the same origin of lunar and terrestrial materials that has been supported by the isotopic anomalies of different elements in these two celestial bodies [18]. Therefore, the indigenous water in the Moon could have originated from the same source as the water in Earth. Consequently, when and how this water was incorporated into the Moon have become key to understanding the physical and chemical conditions in the post-impact protolunar disk. The discovery of water in the LMO crystallization products [5] indicates that water may have been partially retained in the protolunar disk during giant-impact formation of the Moon. Alternatively, this indigenous water could have been added into the Moon during the solidification of the LMO at 4.3–4.5 Ga [19]. We discuss these two possibilities below.

**Figure 1.**
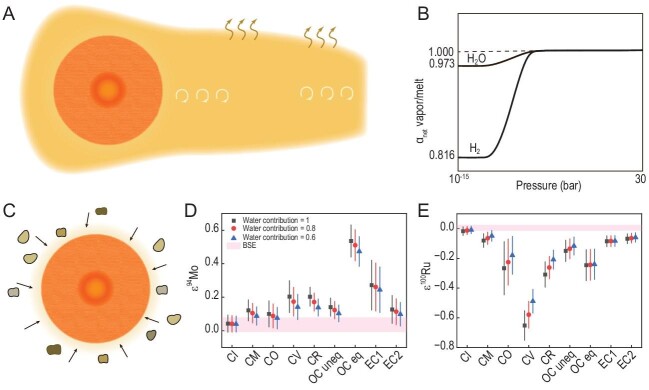
Two scenarios proposed to reconcile the different water contents but similar hydrogen isotope compositions between the early Moon and bulk silicate Earth. (A) An illustration to show protolunar disk surrounded by high-pressure gas media composed of different volatiles, including water. (B) Equilibrium fractionation factor of hydrogen isotopes between melt and vapor at different pressures. The curves show our calculations for two hydrogen species H_2_ and H_2_O, respectively. The pressure of 10^−^^15^ bar is for current lunar atmosphere [23] whereas 30 bar is one estimation of vapor pressure in the protolunar disk after giant impact [20]. Note that the curves between the vacuum and high pressure are for illustrative purpose and require experimental assessment. (C) An illustration to show that water in asteroids accreted into the Moon during impacting the Moon at ∼4.5–4.3 Ga [19]. Isotope anomalies of highly siderophile elements (D) Mo and (E) Ru of the Moon after incorporation of asteroids that delivered water to the early Moon. The symbols square, dot and triangle mean that chondrites have contributed 100%, 80% and 60% of the total lunar interior water, respectively, assuming that the bulk water content in the Moon is 100 ppm. The horizontal bars show the isotope anomalies of bulk silicate Earth. Water contents and isotopic anomalies of carbonaceous chondrites (CI, CM, CO, CV and CR), OC uneq, OC eq, EC1 and EC2 are from the literature [18,19,24]. OC uneq: unequilibrated ordinary chondrites; OC eq: equilibrated ordinary chondrites; EC1: enstatite chondrites with water content of 500 ppm; EC2: enstatite chondrites with water content of 4500 ppm; BSE: bulk silicate Earth. Both illustrations are not to scale.

It is clear that water in the Moon-formation materials could have been evaporated during the giant impact. However, water and volatiles may not have been completely lost during this high-temperature process (Fig. 1A). This evaporation outcome is still consistent with the observations that water and volatiles in the Moon are much more depleted than those in Earth. On the other hand, the initial hydrogen isotope composition in the LMO is similar to that of terrestrial primitive mantle [12,17]. This similarity indicates that evaporation during the Moon formation may not have caused large changes in the hydrogen isotope compositions in the protolunar disk. Therefore, kinetic isotope fractionation may not have been the main process that fractionated the hydrogen isotopes in the early lunar materials from which water was partially evaporated during the Moon formation. On the other hand, hydrogen equilibrium fractionation may have occurred during water evaporation in the protolunar disk. Moreover, giant-impact modeling indicates that pressures in the protolunar disk may have been high [20]. The hydrogen equilibrium fractionation factors between melt and vapor at high pressures could be unity (Fig. 1B). Consequently, the water content and hydrogen isotope composition in early lunar materials after the Moon formation could be consistent with those detected in the LMO products. Nevertheless, further assessment of physical conditions of the protolunar disk is still needed to verify this proposal.

The traditional giant-impact model suggests that all the water could have been lost during the Moon formation [1]. A possible explanation for the discrepancy between the results from this traditional model and the observations of water in the LMO products could be that the water detected in the returned lunar materials was added into the Moon after its formation (Fig. 1C). Water-rich chondrites and a minor amount of cometary material could have accreted into the molten Moon at ∼4.5–4.3 Ga [19,21,22]. However, any later accretion after the Moon formation could alter isotope compositions of other elements in the Moon as well. Assuming that the Moon and Earth have the same isotopic anomalies of Mo and Ru, we can determine the isotopic anomalies of these two highly siderophile elements in the Moon after addition of different chondrites as contributors of 100, 80 and 60 ppm water (Fig. 1D and E). Our calculations demonstrate that the isotope anomalies in the Moon after the late accretion of chondrites may differ from those of bulk silicate Earth (Fig. 1D and E). This disparity may challenge there being the same material sources for the Moon and Earth. Note that the isotope anomalies of Mo and Ru have not yet been determined for bulk silicate Moon [25]. Nevertheless, this discrepancy shows that any later accretion model may need to assess isotope compositions of different elements in lunar materials. This assessment can verify the second proposal.

In summary, the amount of water in the Moon is less than that of bulk silicate Earth but it is believed to have originated from the same source. Two scenarios have been proposed to explain how this indigenous water was incorporated into the Moon. Water may have been partially retained in the protolunar disk during the Moon formation (Fig. 1A). Equilibrium fractionation during water evaporation may have been the primary process that fractionated hydrogen isotopes in the protolunar disk (Fig. 1B). Alternatively, this observed water in the lunar interior could have accreted into the Moon during its molten stage at ∼4.5–4.3 Ga (Fig. 1C). To test these two hypotheses, three different types of new data may be required, including accurate water content and hydrogen isotope composition of the early Moon, isotopic anomalies of siderophile elements in the Moon and equilibrium fractionation factors of hydrogen isotopes at different pressures. Nevertheless, the evaluation of these two proposals could constrain the physical and chemical conditions in the protolunar disk during the Moon formation.
